# Linguistic diversity and theory in psychological science

**DOI:** 10.3389/fpsyg.2026.1866037

**Published:** 2026-10-06

**Authors:** Kenny R. Coventry

**Affiliations:** School of Psychology, University of East Anglia, Norwich, United Kingdom

**Keywords:** crises, grand challenge, linguistic diversity, theory development, universals

## 1 Introduction

When it comes to understanding the human mind and behavior, theory is often generated from and tested via samples that are not representative of the population as a whole. Over 20 years ago, this problem was brought into sharp focus with a number of authors calling for research in the cognitive and psychological sciences to be more aware of diversity and representativeness (e.g., Atran et al., 2005; Norenzayan and Heine, 2005; Rozin, 2001)—a point also more recently recognized in comparative cognition (Andrews and Monsó, 2025). “WEIRD”—Western, Educated, Industrialized, Rich, Democratic (Henrich et al., 2010)—was coined as an acronym to raise awareness that most findings in psychological science and related fields are gleaned from studies with limited participant pools. A decade later, the extent to which such concerns have resulted in changes to practice can be questioned (e.g., Apicella et al., 2020), and a coherent framework to embed linguistic and cultural diversity in psychological theorizing and empirical testing has yet to be established.

In tandem with the recognition of a problem with representativeness in psychological science, two other major issues have emerged as ongoing concerns. First, there is the “reproducibility crisis,” pulling together a range of methodological and statistical issues, including worries regarding sample sizes, statistical reporting, questionable research practices (e.g., *P* hacking) and publication bias (e.g., Open Science Collaboration, 2015). Second, a range of concerns have been expressed regarding the very nature of theorizing in the psychological sciences. Some have drawn attention to the lack of specificity of theories as one of the origins of the replication crisis itself, calling for more formal theories to be *de rigueur* in the discipline (e.g., Oberauer and Lewandowsky, 2019; van Dongen et al., 2025), while others point the finger at the absence of an overarching theory formulation framework that may help to constrain theories to become more appropriately testable (e.g., van Rooij and Baggio, 2021), akin to the “levels of explanation” approach proposed by Marr (1982).

In this article I consider how these three major concerns interconnect in consideration of one of the most essential elements of WEIRDness—the failure to embrace linguistic diversity in both theoretical and empirical psychological science.

## 2 Linguistic diversity and representativeness

There are over 7,000 spoken and signed languages across the globe (Ethnologue, 2026), In contrast to the assumption from generative linguistics that languages share universal features (e.g., Chomsky, 1986; Pinker, 1994), a body of (largely) anthropological linguistic studies has shown that languages vary in significant ways, with diversity found at almost every level of linguistic organization (Evans and Levinson, 2009). For example, languages differ across dimensions including sound systems, phonology, morphology, grammar, orthography, and semantics, leading Evans and Levinson (2009) to conclude that diversity *is* the most remarkable property of language. If the goal of the cognitive and psychological sciences is to identify and study universal psychological processes, akin to Newton's universal laws of physics (Kroupin et al., 2025), then such linguistic diversity may challenge the very nature of the focus on universals (see Levinson, 2012 for discussion). In light of this linguistic diversity, one can ask how the cognitive and psychological sciences are doing prior to and since Evans and Levinson (2009) in terms of sampling speakers of diverse languages. The answer is not very well. The cognitive and psychological sciences have been dominated with publication in English and testing of English-speaking participants. For example, Arnett (2008) noted that the vast majority of articles sampled from each of six major (APA) psychology journals (across disciplines) within the preceding 5-year period reported data from English-speaking samples (ranging from 74% to 91% of articles across the six journals). Moreover, these English-speaking samples were from just five countries: United States, UK, Canada, Australia, and New Zealand. Thalmayer et al. (2021) note that this state of affairs has barely changed in the intervening 10-year period, with a continued preponderance of samples (and authors) from English-speaking countries.

This “Anglocentric” bias may partly be due to the fact that English is spoken by a large percentage of the world's population (around one sixth speak English to a degree of proficiency; Ethnologue), as well as the continued dominance of psychology research in English speaking countries (Thalmayer et al., 2021). If English can be regarded as prototypical of the world's languages, this bias might not be such a problem. But as Blasi et al. (2022) point out, English is atypical of the world's languages with respect to many key properties, including its writing system, grammaticalized categories, morphology, phonology and syntax (see Blasi et al., 2022 for discussion).

This Anglocentric bias is also important for the generation of theory in the psychological and cognitive sciences, given the dominance of psychologists working in English (Arnett, 2008; Thalmayer et al., 2021). A lack of awareness of linguistic diversity can bring with it the assumption that the distinctions in one's language are universal categorical distinctions when they are not, affecting the construction of theory.

Indeed, there are many cases where central psychological theoretical constructs may be a direct reflection of the language of the psychologists doing the theorizing rather than universal concepts sitting outside of language (Brick et al., 2022; Wierzbicka, 2013). For example, it is tempting to think that psychology is the science of the “mind” and that “mental life” is a uniform concept across languages and cultures. But as Weisman et al. (2021) show, although it can be argued that a mind-body distinction may be universal, exactly how components of mental life (especially social-emotional components) are conceptualized differs substantially across languages and cultures (see also Lillard, 1998). To give a second example, the idea that there is a set of basic emotions with privileged status in human mental life and social interactions (e.g., as proposed by Ekman, 1992) has been challenged on the basis that the “enterprise is bedeviled by an overreliance on English affective terms” (Ortony, 2022, p. 41). Wierzbicka (1999) discusses the failure of high-frequency English “basic” emotion words to translate directly into other languages, noting for example that Russian does not have words that mean the same as “sadness” and “anger” in English. Thus, at the broadest level of theory, English-speaking psychologists make assumptions about central constructs that are not universally lexicalized/conceptualized across languages.

Moreover, there is much evidence that the language one speaks is associated with behavioral differences across a broad range of domains. Three examples will make this clear. First, Chen (2013) investigated the relationship between the grammatical encoding of past and future in languages (future time reference) and a range of societal-level behaviors, finding that societies with languages that grammatically associate the future and the present (e.g., today it rains; tomorrow it rains) are 31 percent more likely to have saved money in any given year, have accumulated 39 percent more wealth by retirement, are 24 percent less likely to smoke, are 29 percent more likely to be physically active, and are 13 percent less likely to be medically obese, compared to societies with languages that do not associate past and future (e.g., today it rains; tomorrow it *will* rain). Second, languages encode number in different ways (see Xu et al., 2020), with some languages possessing only approximate number terms (akin to vague quantifiers, such as *few, some*, and *lots of*, in English) while others have exact (recursive) number systems (e.g., English). It has been shown that the Pirahã, an Amazonian people with an absence of exact number terms in their language, are inaccurate in remembering large quantities compared to English speakers (Frank et al., 2008; Gordon, 2004). Third, color vocabularies exhibit considerable cross-linguistic variation, and a body of evidence indicates that speakers of different languages show different patterns of color discrimination (see Lupyan et al., 2020 for a review), with effects emerging as early as 100 ms after viewing a color (e.g., Thierry et al., 2009). While the relationship between language, cognition and culture is clearly complex (see Blasi et al., 2022 for discussion), the evidence that the language one speaks influences a wide range of behaviors is compelling.

So most studies in psychology remain dominated by testing English speakers. However, it is important to note that the scientific study of language itself is not immune to this Anglocentric bias. Berghoff and Bylund (2025) document a lack of diversity of languages in a sample over 5,500 published psycholinguistic studies over a 20-year period, with 45% of published studies on English, and 76% overall comprising data from English and only nine other languages. Another key concern is that linguistics itself in many quarters lacks the methodological rigor that psychologists have been aspiring to for many years. The normative model in anthropological linguistics is to work with a small number of linguistic informants to glean information about language. This makes an implicit assumption that speakers of a language may make the same distinctions, and therefore that a few speakers of a language are representative of the population from which they are drawn. This assumption has recently been challenged with a large-scale study of spatial demonstratives (e.g., *this/that/here/there*) across 29 languages. Coventry et al. (2023) tested the production of demonstratives experimentally with sample sizes with sufficient power to quantify variation both between and within languages. For all 29 languages tested, significant within-language variation was found, challenging the view that generalizations about a language can be made from a handful of linguistic informants. Variation within a population is something that is often overlooked in typical anthropological studies with small numbers of participants, and large-scale cross-linguistic experimental studies are relatively rare (cf. Majid et al., 2018; Coventry et al., 2023).

## 3 The way forward? Linguistic diversity and psychological science

In this last section, I briefly consider how linguistic diversity can be embraced by psychological science in light of the three crises identified at the start—diversity/representativeness, reproducibility, and theory construction in psychology.

### 3.1 Diversity/representativeness and reproducibility

One can ask what can be done to improve consideration of linguistic (and cultural) diversity in both psychological scientific theory and empirical testing. If the goal of the cognitive and psychological sciences is to identify what is and is not universal for our species, psychological science should move to conducting large-scale (suitably powered) studies across multiple languages and cultures as a matter of course. Practically this is challenging, given that research funding is still largely nationally determined, and the contact base for most researchers does not include extensive networks of researchers working on other languages and cultures. However, the Open Science Collaboration and the Many Babies Project (https://manybabies.org/) are examples of large-scale collaborative networks of researchers addressing issues of reproducibility across multiple labs, and some examples of such cross-linguistic collaborations across large numbers of languages and teams (e.g., Majid et al., 2018; Coventry et al., 2023) show that such endeavors are possible. Funders would do well to encourage more collaborative projects at the required scale. Of course, in doing so, multiple sites collecting data across languages also plays to several of the key concerns identified in the reproducibility crises. Namely, establishing if phenomena generalize beyond specific samples and if the methods are reproducible across diverse populations.

At a smaller scale, Majid (2023) suggests that culturally informed, theoretically motivated sampling can provide a useful model for research where larger scale sampling is not possible. To do so, researchers can select cultures and languages that are maximally distinct for the research question at stake as a (more modest) test of universals, with iterative testing/replication across other languages that are similarly (maximally) distinct.

Practically many researchers lack the knowledge of other languages and cultures to be able to select samples that are maximally distinct. Part of the issue here is that psychologists are simply not adequately trained in linguistic and cultural diversity. National and international bodies representing the discipline need to encourage university/school programs for undergraduates and graduates to include training in this area.

Finally, there is a need for journals and learned bodies to develop guidelines for reporting and to improve publication practices to encourage diversity in sampling. This should include explicit declarations regarding samples and assumptions at all stages of reporting. Blasi et al. (2022) note that English-language researchers do not have to mention (in titles, abstract, or limitations in discussions) that their findings pertain only to English, but this is not the case for researchers reporting results for other languages. Moreover, Kroupin et al. (2025) call for an explicit statement regarding the environment/group tested and whether this group is universal or group specific. Publication outlets should also prioritize publishing findings from diverse samples recognizing the importance of such endeavors to theory development in the psychological and cognitive sciences.

### 3.2 Theory construction

Linguistic diversity must also be considered in relation to the whole research process, and not just in terms of testing across diverse languages and cultures. At the level of construction of a theory, a key element that is often missed is the extent to which the construction of a theory is bound by the language in which it is constructed, and relatedly if a theory can withstand scrutiny across language contexts. I provided some examples earlier, but examples are pervasive across the spectrum of psychological science subdisciplines. Ideally, at the point of construction of a theory one can ask if the constructs emerging may be language specific or if they are robust across languages. Perhaps the easiest way in which this can be done is to interact with psychologists working in diverse languages where possible. The idea from Majid (2023) for culturally informed, theoretically motivated sampling can be extended to the construction of research teams, where theory is constructed by sampling theorists working in diverse languages. This comes back to the dominance of English and only a few other languages as the language of psychological science—the push for diversity and representativeness of researchers at conferences and in journal editorial boards, etc. becomes central to questioning if the constructs can be truly universal. At the very least, theories need to acknowledge possible constraints from the language in which theories are constructed.

## 4 Conclusions

A tacit assumption in the psychological and cognitive sciences is that languages are essentially all the same. As Pinker put it “a visiting Martian scientist would surely conclude that aside from their mutually unintelligible vocabularies Earthlings speak a single language” (Pinker, 1994, p. 232). This assumption has been challenged with evidence of substantive variation across all levels of language structure. If the goal of cognitive and psychological sciences is to search for universal constructs, embracing linguistic diversity both in theory and practice is essential for advancement of these disciplines.
